# Opportunities and challenges for identifying undiagnosed Rare Disease patients through analysis of primary care records: long QT syndrome as a test case

**DOI:** 10.1007/s12687-024-00742-7

**Published:** 2024-10-15

**Authors:** William Evans, Ralph K. Akyea, Alex Simms, Joe Kai, Nadeem Qureshi

**Affiliations:** 1 Primary Care Stratified Medicine (PRISM), Centre for Academic Primary Care, School of Medicine, University of Nottingham, Applied Health Research Building [42], University Park, Nottingham, NG7 2RD UK; 2 Department of Cardiology, Leeds Teaching Hospital NHS Trust, Leeds, UK

**Keywords:** Prolonged QT interval, Long QT syndrome, Genetics, Clinical prediction, Rare disease, Primary care

## Abstract

**Background:**

Patients with rare genetic diseases frequently experience significant diagnostic delays. Routinely collected data in the electronic health record (EHR) may be used to help identify patients at risk of undiagnosed conditions. Long QT syndrome (LQTS) is a rare inherited cardiac condition associated with significant morbidity and premature mortality. In this study, we examine LQTS as an exemplar disease to assess if clinical features recorded in the primary care EHR can be used to develop and validate a predictive model to aid earlier detection.

**Methods:**

1495 patients with an LQTS diagnostic code and 7475 propensity-score matched controls were identified from 10.5 million patients’ electronic primary care records in the UK’s Clinical Practice Research Datalink (CPRD). Associated clinical features recorded before diagnosis (with *p* < 0.05) were incorporated into a multivariable logistic regression model, the final model was determined by backwards regression and validated by bootstrapping to determine model optimism.

**Results:**

The mean age at LQTS diagnosis was 58.4 (SD 19.41). 18 features were included in the final model. Discriminative accuracy, assessed by area under the curve (AUC), was 0.74, (95% CI 0.73, 0.75) (optimism 6%). Features occurring at significantly greater frequency before diagnosis included: epilepsy, palpitations, syncope, collapse, mitral valve disease and irritable bowel syndrome.

**Conclusion:**

This study demonstrates the potential to develop primary care prediction models for rare conditions, like LQTS, in routine primary care records and highlights key considerations including disease suitability, finding an appropriate linked dataset, the need for accurate case ascertainment and utilising an approach to modelling suitable for rare events.

**Supplementary Information:**

The online version contains supplementary material available at 10.1007/s12687-024-00742-7.

## Introduction

In the European Union a disease is classified as rare if it affects fewer than 1 in 2000 persons (Moliner and Waligora 2017), 70% of which are genetic diseases (Nguengang Wakap et al. 2019). Although individually rare they are collectively common, affecting 3.5 -5.9% of the population (Nguengang Wakap et al. 2019). Diagnosis of these disorders is often challenging, patients frequently experience a difficult and protracted route to diagnosis, with some never receiving an accurate diagnosis (Gainotti et al. 2018). This “diagnostic odyssey” is associated with additional morbidity, missed opportunities for treatment, inappropriate and often ineffective treatments and costly investigations (Evans and Rafi 2016). Addressing this delay is a key priority of rare disease health policymakers (Department of Health and Social Care 2021; Khosla and Valdez 2018; Moliner and Waligora 2017).

Rare disease decision support systems have been developed to aid diagnosis (Liévin et al. 2023; Ronicke et al. 2019). Most are designed to be reactive, they require the clinician to suspect a rare disease and then utilise the tool to refine the differential diagnosis. A shortcoming of this approach is that it will miss patients for whom a rare disease diagnosis is not suspected. An attractive approach to address this shortcoming is to use routinely collected healthcare data to “flag” or stratify patients at risk of an undiagnosed disease at a population level. There is growing potential for this approach as electronic health records (EHRs) are more widely adopted and records from different clinical settings linked together. In the UK the primary care EHR has the potential to be utilised for such an approach. Over 90% of individuals are registered with a primary care practice and EHRs have been established for many years with coded data for some patients extending over more than two decades (Goldacre 2022). This coded data has been used in a pilot project that flagged patients at risk of a number of rare diseases based on coded clinical features in their EHR (Buendia et al. 2022).

Developing multivariable prediction models for rare genetic diseases is a natural extension of the development of such models for a growing range of diseases and scenarios (Collins et al. 2024). There are, however, specific challenges in rare disease, most notably the small number of patients affected by each disease, but also that rare disease are frequently highly heterogeneous, and the diagnostic coding for some rare disease may be limited in the EHR.

Long QT syndrome (LQTS), first described in 1957 (Jervell and Lange-Nielsen 1957), are a group of inherited cardiac arrhythmia that occur in the absence of structural heart disease and predispose patients to syncope and sudden cardiac death (Schwartz et al. 2012; Schwartz and Ackerman 2013). LQTS is one of several causes of a prolonged QT interval, an electrocardiogram finding associated with potentially fatal arrhythmias, other causes include myocardial ischaemia, electrolyte disturbances and medications.

The prevalence of LQTS in live births is approximately 1 in 2000 (Schwartz et al. 2009) and is the likely aetiology in a substantial number of sudden cardiac death in the young (Ackerman et al. 2016).

LQTS has been associated with 17 different genes, 7 with strong evidence of causality, the 3 most common of which have a clear gene specific phenotype and are described as clinically distinct subtypes (Adler et al. 2020).

Untreated LQTS patients have high rates of cardiac events and mortality (Priori et al. 2003). However, patients still experience a long diagnostic delay, episodes of tachyarrhythmia which are usually self-limiting are frequently misdiagnosed as epilepsy, vasovagal syncope or breath-holding attacks (Schwartz et al. 2012). Early diagnosis is important as beta-blockers, the mainstay of treatment, drastically reduce mortality (Schwartz and Ackerman 2013), whilst implantable cardioverter defibrillators (ICD) are fitted in those at high risk of SCD (Rohatgi et al. 2017; Schwartz et al. 2012). Diagnosis also enables specific lifestyle adjustments, avoidance of medications that further prolong the QT interval and cascade screening of family members (Priori et al. 2015).

While episodes of syncope are regarded as typical of LQTS, there is a lack of substantive evidence on the range of clinical features in LQTS, and how undiagnosed patients may present to primary care.

LQTS was chosen as a suitable exemplar of a rare genetic condition whose identification could be improved by a primary care prediction model for the following reasons. Firstly, early diagnosis is critical, secondly, we expect patients to have clinical features in their primary care record preceding diagnosis, and finally, although LQTS is a rare disease it sufficiently common that one would expect there to be sufficient cases in a large primary care research dataset to identify early clinical features, develop and internally validate a prediction model.

## Methods

### Data source

The UK Clinical Practice Research Datalink (CPRD) GOLD is an electronic medical record database with longitudinal data from 1987 to the present. In its entirety, it has 681 UK family physician practices’ data, including 35 million patient lives, of which 15 million patients are currently registered (CPRD 2023). It is considered representative of the general population and used to support the design and implementation of large epidemiological studies (Akyea et al. 2019; Herrett et al. 2015; Khan et al. 2010). This study was approved by the Independent Scientific Advisory Committee for the Medicines and Healthcare Products Regulatory Agency (ISAC Protocol 19_049).

### Study design and population

1495 patient records with a diagnostic code for Long QT Syndrome (LQTS) were identified from CPRD (total number of patient records 10.5 million). LQTS patients were identified by the presence of one or more of the following diagnostic codes CTV3 Read codes: Long QT syndrome (X202j/G56y500); Andersen-Tawil Syndrome (LQTS type 7) (Xagdx); Romano Ward Syndrome (G56y200); Jervell and Lange-Nielsen Syndrome (G56y300) (*Read Codes*, n.d.). The index date was defined as the first date that one of these codes was documented in the patient’s record.

Patients were eligible for enrolment if registered with their primary care practice for at least 12 months. Data was collected from the time their practice’s data was deemed to meet CPRD’s data quality standards (CPRD 2023) until the date of final data extraction in July 2018.

Propensity score (using gender, age, BMI, smoking status and ethnicity) was used to match each case to five (5) controls from the same practice, no other limitations, such as other cardiac conditions, were placed on the control population. Propensity scores allow for observational studies to mimic particular characteristics of a randomized controlled trial by balancing the distribution of observed baseline covariates between groups (Austin 2011).

Following a review of the published literature and discussion with colleagues and experts, a series of potential and hypothesised clinical features that may occur in advance of an LQTS diagnosis were created (Supplementary Material 1 and 2) and mapped to the appropriate diagnostic codes. The data set was searched to identify these clinical features appearing prior to the index date, for both the LQTS cases and the equivalent age for each of the cases’ five (5) matched controls. We used multiple imputation to create 10 imputed datasets for missing values for BMI, blood pressure, pulse rate, potassium and calcium blood levels, using chained equations and combined the measurements via Rubin’s rules to develop a final estimate (Marshall et al. 2009; Royston and White 2011).

Findings were reported using the guidance in the Transparent Reporting of a Multivariable Prediction Model for Individual Prognosis or Diagnostic (TRIPOD) statement (Collins et al. 2015).

### Statistical analysis

Logistic regression models were used to incorporate all pre-specified clinical features with a known or suspected association with LQTS identified from the literature. We used backwards regression modelling, removing one feature in each round, to optimise the model comparing Bayesian information criterion (BIC), Ataike information criterion (AIC), Area under the ROC curve (AUC) and calibration plots with each iteration. The optimum final model for predicting the outcome, diagnosis of LQTS, was determined by achieving the minimum BIC and AIC, and therefore minimising over-fitting, had its performance evaluated by AUC and calibration. AUC indicates the probability that for a randomly selected pair, one with and one without LQTS, the LQTS patient has a higher predicted risk, with 1.00 indicating perfect discrimination and 0.50 no discrimination (Vickers and Elkin 2006). Multiple iterations of the model were performed using both logarithmic transformations of continuous clinical features and calculating the fractional polynomials for these same variables to improve model calibration. All analyses were performed with Stata 15.1 (StataCorp LP).

### Validation analysis

Bootstrapping was performed as described by Harrell et al. (1996). The data set was repeatedly resampled to produce 200 replicated sets, each the same size as the original. The model was fitted to each of these 200 data sets, with each fitted model then applied to the resampled data from which it was generated as well as the original data set. The mean AUC from the refitted model for each of these 200 data sets was then calculated and the difference between this and the AUC of the original data set’s model was calculated. The original AUC minus this difference was then calculated to give an Optimism AUC with 95% confidence intervals.

### Sensitivity analysis

The following sensitivity analyses were performed:

A re-analysis of the associations between clinical features and a diagnosis of LQTS in sub-groups of patients diagnosed at less than 45, 40 and 35 years of age, to allow for miscoding of older patients with LQTS, many of whom may have a prolonged QT interval of other causes. The discrimination of the predictive model derived from the whole data set was assessed by AUC in these subgroups.

A further analysis was performed in a more tightly phenotyped subgroup of cases and their controls, that following their diagnosis have a record of a beta-blocker prescription and/or an implantable cardioverter defibrillator (ICD).

Further iterations of the model were performed excluding four of the clinical features: mitral valve disease, hypertension, coronary artery disease (clinical issues that may lead to a prolonged QT interval that isn’t caused by LQTS) and ethnicity (as this was poorly recorded).

## Results

### Baseline characteristics (see Table 1)

There was a total of 8970 individuals in this study. 1495 patients with an LQTS diagnostic code and 7475 controls. In our sample, most cases were female (67%). 74% of cases were either normal or overweight (BMI >/= 18.5 < 30 kg/m2). Most were non-smokers (59%). Ethnicity was poorly recorded, not defined for approximately half of the patients and if recorded overwhelmingly white (94% of those with ethnicity declared). Some continuous variables were poorly recorded most notably pulse and calcium levels in the control population (Supplementary material 5). The median age of receiving a diagnostic code in their EHR was 54.1 years (IQR 39.3, 69.2). When this was restricted to those who were prescribed a betablocker at the time of diagnosis (defined as upto 60 days before the first date of LQTS diagnosis) or at any point following diagnosis (*n* = 293) the median age was 44.4 (IQR 29.9, 61.0).

**Table 1:** Baseline characteristics

Characteristics		Cases LQT	Controls
**Numbers (%)**		1495 (16.67)	7475 (83.33)
**Gender (male)**	*n* (%)	495 (33.11)	2618 (35)
**Gender (female)**	*n* (%)	1000 (66.89)	4650 (65)
**Age at diagnosis**	mean (sd)	54.10 (19.41)	
**BMI (kg/m**^**2**^)	mean (SD)	26.8 (5.65)	26.4 (4.83)
**Smoking status**	no data *n*(%)	109 (7.29)	1538 (20.58)
	smoker *n*(%)	319 (21.34)	1664 (22.26)
	non-smoker *n*(%)	882 (59.00)	3445 (46.09)
	ex-smoker *n*(%)	185 (12.37)	828 (11.08)
**Ethnicity**	white *n*(%)	706 (47.22)	2316 (30.98)
	non-white *n*(%)	42 (2.8)	188 (2.5)
	Unknown *n*(%)	747 (49.97)	4971 (66.50)

**Table 2:** Clinical characteristics derived from the univariate model of LQTS

		Cases LQT	Controls	*P* value
**Numbers**		1495 (16.67)	7475 (83.33)	
**Cardiovascular**				
Systolic BP	Mean (SD)	130.84 (20.02)	128.22 (18.62)	
Diastolic BP	Mean (SD)	77.67 (11.47)	76.24 (11.18)	
Diagnosis hypertension	Recorded *n* (%)	463 (30.97)	1101 (14.73)	< 0.001
Pulse	Mean (SD)	76.08 (13.617)	75.76 (12.64)	
Tachycardia on pulse (mean > 100)	Recorded *n* (%)	25 (1.67)	52 (0.70)	< 0.001
Bradycardia on pulse (mean < 60)	Recorded *n* (%)	54 (3.61)	109 (1.46)	< 0.001
Diagnosis aortic valve disease	Recorded *n* (%)	22 (1.47)	46 (0.62)	< 0.001
Diagnosis mitral valve disease	Recorded *n* (%)	25 (1.67)	25 (0.33)	< 0.001
Diagnosis palpitations	Recorded *n* (%)	195 (13.04)	308 (4.12)	< 0.001
Diagnosis heart failure	Recorded *n* (%)	43 (2.88)	75 (1.00)	< 0.001
Diagnosis coronary arterial disease	Recorded *n* (%)	152 (10.17)	337 (4.51)	< 0.001
Diagnosis atrial fibrillation (AF)	Recorded *n* (%)	104 (6.96)	128 (1.71)	< 0.001
**Subfertility/Gynaecological**				
Diagnosis amenorrhoea	Recorded *n* (%)	51 (3.41)	185 (2.47)	0.039
Diagnosis Stillbirth/miscarriage	Recorded *n* (%)	60 (4.01)	197 (2.64)	0.004
**Musculoskeletal (MSK)**				
Diagnosis rheumatoid arthritis	Recorded *n* (%)	20 (1.34)	58 (0.78)	0.033
**ENT/Respiratory**				
Diagnosis Asthma	Recorded *n* (%)	184 (12.3)	801 (10.7)	0.072
**Neurological**				
Diagnosis stroke/ TIA	Recorded *n* (%)	36 (2.41)	123 (1.65)	0.041
Diagnosis epilepsy	Recorded *n* (%)	40 (2.68)	88 (1.18)	< 0.001
Diagnosis migraine	Recorded *n* (%)	19 (1.27)	50 (0.67)	0.015
Diagnosis dizziness	Recorded *n* (%)	265 (17.73)	699 (9.35)	< 0.001
Diagnosis collapse	Recorded *n* (%)	170 (11.37)	361 (4.83)	< 0.001
**Biochemistry**				
**Calcium**				
Number with recorded calcium level		560 (37.45)	2084 (28.88)	
Mean calcium	Mean (SD)	2.317 (0.1214)	2.330 (0.137)	
Hypocalcaemia on mean reading		64 (4.281)	118 (1.579)	< 0.001
**Potassium**				
Number with recorded potassium level		1065 (71.24)	2912 (38.96)	
Mean Potassium	Mean (SD)	4.294 (0.490)	4.380 (0.487)	
**Other**				
Diagnosis Irritable bowel syndrome	Recorded *n* (%)	130 (8.70)	274 (3.67)	< 0.001

### Multivariable modelling

**Fig. 1:**
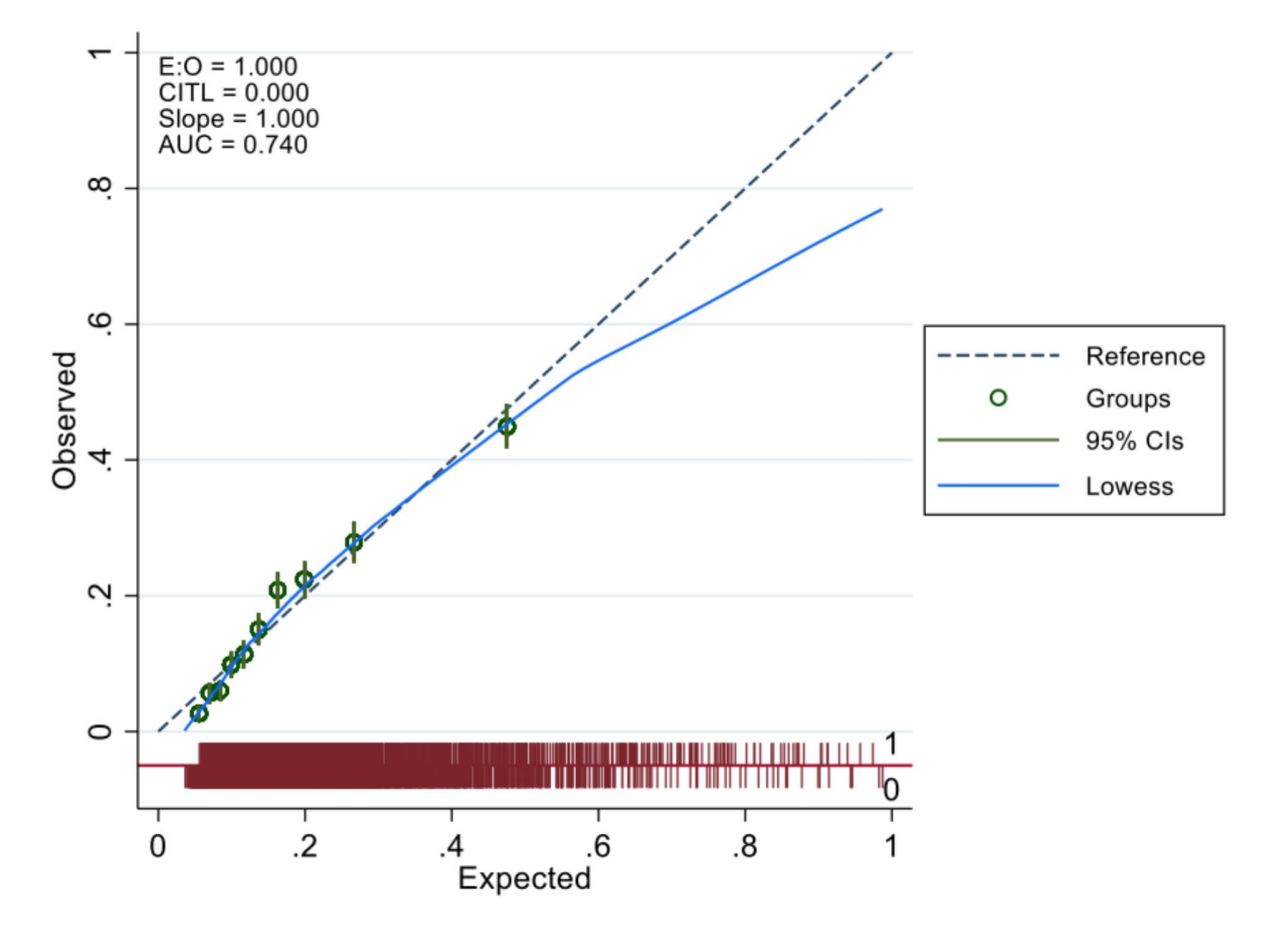
Assessing model calibration comparing expected vs. observed risks. *The dashed line represents perfect calibration*,* the model’s predicted probabilities exactly match the observed probabilities. The Lowess curve indicates that the model is well calibrated until 0.5 expected probability after which it begins to slightly over-predict risk*

**Fig. 2:**
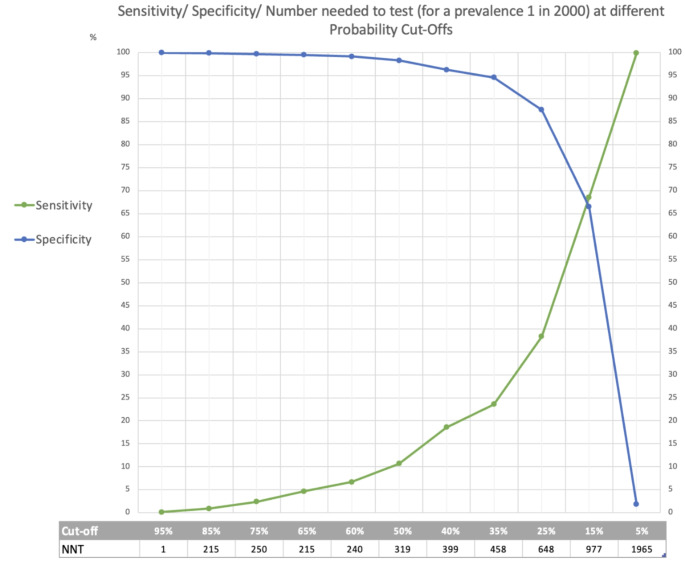
Threshold analysis plot: Sensitivity/ Specificity/ Number needed to test at different probability cut-offs. *The table shows the sensitivity and specificity of the model at different probability cut-offs. The number needed to test (NNT) indicates the number of patients identified by the model that would need to be investigated to identify one person with LQTS*,* at a prevalence of 1 in 2000*

The optimum model, incorporating the clinical features in Table 3, had an AUC of 0.74 (95% CI, 0.73, 0.75). The overall calibration slope of the model was 1.0 (Fig. 1), with good calibration until above an expected probability > 0.5, where the model then tended slightly to over-predict risk. Previous iterations from the model development can be found in supplementary materials (Supplementary material 4). The performance of the model, sensitivity, specificity, and number needed to test (NNT), the number that needed to be flagged by the model to identify one case, for an LQTS prevalence of 1 in 2000 is demonstrated in Fig. 2.

A subset of clinical features was combined to calculate the odds ratio (OR) of a grouping of clinical features included in the model: a female under the age of 45 with irritable bowel syndrome and: dizziness, and/or collapse and/or palpitations OR 5.06 (95% CI, 2.75, 9.28).

**Table 3:** Clinical features incorporated into the final multivariable analysis

Clinical Variable	Odds Ratio (95% CI)	Std. Err.	Beta Coefficient (95% CI)
Diagnosis Hypertension	1.64 (1.42, 1.91)	0.124	0.496 (0.348, 0.644)
Average pulse category: tachycardia(> 100 bpm)/ normal/ bradycardia (< 60 bpm)	1.23 (1.03,1.47)	0.109	0.208 (0.034, 0 0.382)
Diagnosis of bradycardia	3.00 (1.62, 5.56)	0.943	1.10 (0.483, 1.71)
Diagnosis of tachycardia	1.49 (0.99, 2.26)	0.316	0.405 (-0.00819, 0.818)
Diagnosis of Coronary artery disease	1.38 (1.09, 1.74)	0.162	0.320 (0.090, 0.551)
Diagnosis mitral valve disease	2.64 (1.41, 4.91)	0.837	0.969 (0.347, 1.59)
Diagnosis atrial fibrillation (AF)	1.91 (1.41, 2.60)	0.300	0.649 (0.342, 0.957)
Diagnosis palpitations	2.22 (1.80, 2.74)	0.239	0.797 (0.586, 1.01)
Diagnosis dizziness	1.237 (1.04, 1.47)	0.111	0.212 (0.0371, 0.388)
Diagnosis collapse	1.636 (1.32, 2.03)	0.179	0.493 (0.279, 0.707)
Diagnosis epilepsy	1.70 (1.12, 2.56)	0.358	0.529 (0.116, 0.942)
Diagnosis of irritable bowel syndrome	1.78 (1.41, 2.26)	0.215	0.579 (0.342, 0.815)
Ethnicity White Non-White	1.37 (1.29, 1.45)	0.042	0.312 (0.252, 0.372)
Smoking status	1.18 (1.09, 1.26)	0.043	0.163 (0.091, 0.235)
Log BMI^ ^2,3^		.	-16.49 (-23.27, -9.70) 2.45 (1.42, 3.47)
Log Average calcium level ^2,3			-35.85 (-53.96, -17.74) 26.51 (12.34, 40.67)
Log Average Potassium ^3,3			-4.46 (-5.89, -3.02) 5.25 (3.27, 7.24)
Average diastolic BP ^0.5,1			-5.37 (-7.09, -3.65) 0.32 (0.22, 0.42)

### Validation analysis

The bootstrap analysis with 200 repetitions generated a mean AUC of 0.81 (95% CI 0.79, 0.82) a 0.06 difference (improvement) from the original data set’s AUC. This difference was then utilised to calculate the optimism AUC 0.68 (95% CI 0.66, 0.69).

### Sensitivity analysis

The predictive model was re-examined in subgroups of patients with their first LQTS diagnostic code under the age of 45, 40 and 35 years of age. This reduced the numbers to 496 cases and 2500 controls; 387 cases and 1955 controls; and 288 cases and 1476 controls respectively. The baseline characteristics of the under-45 sub-group are included in the supplementary materials and the univariate analysis of the range of clinical features included in this population (Supplementary material 3). The predictive model incorporating the clinical features in Table 3 in this under-45 sub-group had an AUC = 0.72 (95% CI 0.69, 0.74). Further analyses were performed in subgroups under the age of 40 (AUC 0.71, 95% CI 0.68, 0.74) & 35 (AUC = 0.69 95% CI 0.66, 0.73).

Further analyses were performed on a subgroup of cases and their controls who following diagnosis, and therefore outside of the period of analysis, were started on any beta blocker and/or had an ICD implanted (681 cases, 3505 controls) (AUC 0.75, 95% CI 0.73, 0.77); and in those commenced on specific beta blockers - nadolol or propranolol - and/or had an ICD implanted (248 cases, 1290 controls) (AUC 0.75, 95% CI 0.72, 0.78).

A further iteration of the model was performed excluding the following clinical features: diagnosis of mitral valve disease, hypertension, coronary artery disease and record of ethnicity (white non-white) (AUC 0.71, 95% CI 0.69, 0.72).

## Discussion

### Principal findings

To our knowledge, this is the largest observational study of LQTS in the general primary care population now available. This has confirmed some expected clinical features: collapse, dizziness, palpitations and epilepsy; but also highlighted less expected clinical associations: irritable bowel syndrome, mitral valve disease and hypertension. We have also found these features can be incorporated with others into a clinical prediction model with an AUC of 0.74, indicating a 74% probability that the risk score would be higher for someone who would develop LQTS than someone who would not. Using a more tightly phenotyped cohort in sensitivity analyses, by limiting analysis to patients diagnosed at a younger age and also in those who were subsequently started on LQTS treatments, demonstrated similar AUC values as the main analysis.

### Comparison with other literature/studies

Current understanding of the clinical features of LQTS is largely based on specialist registries (Ergül et al. 2021; Rohatgi et al. 2017), the largest having more than 2000 subjects. These datasets are from patients in hospital settings focussed on outcomes and treatment effects. Features before diagnosis, if present, have been collected at enrolment and focussed on cardiac outcomes, such as episodes of syncope; aborted cardiac arrest (ACA); and SCD in family members (24). Despite the richness of LQTS registry data, their focus is on the cardiovascular outcomes following diagnosis rather than how this disease may present earlier in its trajectory. For example, the 1-2-3_LQTS_Risk model stratifies patients with known LQTS for their risk of a life-threatening arrhythmia to inform management (Mazzanti et al. 2022).

The data from this large primary care study confirms the following associations from smaller studies: women outnumber men 2 to 1, consistent but more pronounced than previous studies (Locati et al. 1998; Zareba 2019); an association with irritable bowel syndrome, 8.70% of LQTS patients versus 3.67% of controls, although the magnitude of difference is greater than expected, as only certain LQTS subtypes are associated with functional gastrointestinal disorder (Beyder and Farrugia 2016; Locke et al. 2006). The higher rates of mitral valve disease have previously been seen in LQTS. In the pre-genetic era international LQTS registry, when diagnosis was based on clinical criteria alone, 9% of patients had a documented mitral valve prolapse. However this may have represented misdiagnoses of LQTS as mitral valve prolapse is known to be associated with a prolonged QT interval in the absence of LQTS (Moss et al. 1985). LQTS patients are also known to have a higher prevalence of atrial fibrillation (AF) than the general population (Johnson et al. 2008).

### Strength and limitations

The findings represent the real-world experience of primary care patients, with the model based on clinical variables routinely collected in primary care as part of standard care. The cohort was derived from a high-quality primary care database, which is broadly representative of the general population of the UK, and a large sample size (1495 cases) given the rarity of LQTS. We performed a robust internal validation of the model by bootstrapping across 200 repetitions, and in the sensitivity analyses the model performed comparably well in more tightly phenotyped groups: younger subsets of patients and a subset subsequently commenced on treatment for LQTS.

We do however recognise the following limitations in our study. Most significantly the misclassification of LQTS cases, cases were defined by the presence of an LQTS diagnostic code in their EHR. There was no facility to confirm the accuracy of this with either electrocardiogram or molecular test result. The age profile, the median age of diagnosis significantly older than anticipated, and the relatively small proportion of cases that after diagnosis are recorded as receiving a beta-blocker (in particular nadolol or propranolol), which one expect most patients with LQTS to receive, or an ICD, suggests that a sizeable proportion of cases with an LQTS diagnostic code may not have LQTS. This misclassification may be particularly exacerbated in this rare disease by the fact that the diagnostic term Long QT syndrome, includes the ECG finding, a finding that isn’t unique to this genetic rare condition but also associated with other causes. This may have an impact on the validity of the model, however those misclassified are still likely to have a prolonged QT interval, even if another aetiology, and would still be at risk of tachyarrhythmias and sudden cardiac death, so early identification and evaluation of all these patients is important.

It is also possible that LQTS cases in advance of their diagnosis code being recorded may have greater clinical involvement, recording of clinical features and coded entries, reflecting clinical contact rather than a real difference in frequency of these features.

Bias due to under-recording of diagnosis and other missing data is acknowledged, a limitation shared with other large databases and population studies. The impact of missing data has been mitigated by using multiple imputation (Hippisley-Cox et al. 2017; Kaasenbrood et al. 2016). The control population was propensity-matched, which enables the distribution of observed baseline covariates to be balanced between cases and controls, however, we did not exclude patients with certain comorbidities, such as ischaemic heart disease from the control group. LQTS is a rare disease, therefore undiagnosed patients are unlikely to feature significantly in the control group.

### Clinical implications & research recommendations

The prevalence of LQTS identified in this primary care population is much lower than the expected published estimates, this is even more marked if a sizeable proportion of cases had received their diagnostic code inappropriately. This highlights the significant under-diagnosis of this condition, important as undetected LQTS patients experience significant morbidity and mortality. Further, although misclassification may have given a more exaggerated impression, late diagnosis is demonstrated by the age at which LQTS coded in the EHR (Median 54 years). Greater clinical awareness of the range of expected and less expected clinical features found among LQTS patients is needed, enabling earlier detection by lowering clinicians’ index for suspicion and threshold for further investigation. For example, women with irritable bowel syndrome and dizziness may be under-investigated in clinical practice, but we found them to be at a significantly increased risk of LQTS. Further research to explore if this findings is confirmed in other datasets is recommended.

Despite the relative rarity of LQTS, the predictive performance is comparable to established clinical risk models for much more common cardiovascular disease (Hippisley-Cox et al. 2017; Kaasenbrood et al. 2016), demonstrating the potential of this approach for developing clinical prediction tools from primary care data for other rare diseases.

Further research could include external validation of this model in a cohort where the diagnosis can be corroborated with ECG or molecular findings.

Following validation, the model could be used as a ‘pre-screening’ tool to identify at risk patients for recall and further investigation. With the next step for those recalled a targeted family history, enquiring there is personal history of syncope and its trigger, and performing a resting ECG. Further investigation, with exercise and/or 24 h ECG and molecular testing; could then be performed dependent on their answers and ECG finding, using an existing ECG risk calculator (Vink et al. 2018), and the LQTS probability or ‘Schwartz-score’ (Schwartz and Ackerman 2013). At what level the model should ‘flag’ patients for recall is dependent on several things, but perhaps most importantly what resources are available and the impact on those flagged who do not have disease. The challenge is that as LQTS is rare the number of patients that would need to be recalled is high. If we compare to thresholds for investigation in cancer, the suspected cancer pathway in the UK uses clinical features that should prompt referral for investigation, with a 3% PPV or NNT equal to 33 or fewer (NICE 2023). In the US breast screening is now recommended for women aged 40–49 years, in this age bracket the number needed to screen to prevent one cancer death is 753 (Myers et al. 2015). In this model if we use a probability cut off of 15%, where both the sensitivity and specificity are approaching 70%, 977 individuals would need to be recalled and further investigated to identify one individual. This would be a significant undertaking and use of resource.

### Implications for other rare diseases

This study demonstrates that prediction models, developed from primary care EHR data, have the potential as a tool to improve diagnosis of other rare condition. It also highlights some key considerations for RD prediction model development grouped under two broad areas: the disease, and the analytical approach.

### The disease

First, there needs to be a clear need for improvement in the path to diagnosis of the RD. Second, the disease should have a sufficient delay in diagnosis to justify endeavours and for patients to have had the opportunity to engage with health services and therefore for relevant health data to be captured in the EHR. Third, one should expect the disease to have features recorded in the dataset used for analysis and in such a way that can be searched for and interrogated, typically coded EHR entries. For example, aggressive paediatric rare diseases are unlikely to have had many health contacts or investigations in primary care, and even if clinical features are captured, it is unlikely that there would be a sufficient length of engagement with primary care health services before diagnosis that could be used to identify the at-risk patient and steer them into the appropriate diagnostic pathway. Fourth, one must be able to confidently define cases, a significant limitation in this study. This starts with the choice of disease, considering ways in which the cases and controls may be incorrectly assigned, and how the disease is coded in the primary care record. For some ultra-rare diseases, there may be insufficient coding refinement to define the exact disease with coding limited to the parent diagnostic term. Consideration should be given to how the diagnosis can be corroborated with other linked data sources, such as specific prescribed medications, recorded pathology/laboratory testing, or procedures. For example, some RD have recommended surveillance with imaging or blood tests, capturing these tests at the standard interval, would enhance the confidence one would have with diagnosed cases in the dataset.

Fifth, one should consider the homogeneity of the disease. Is it more appropriate to target the entire disease, specific subtypes, or a broader approach clustering several similar diseases together? For example, in this study, we defined LQTS as a single clinical entity, despite it being a syndrome with multiple subtypes. If diagnostic coding had allowed, one could have performed an analysis on certain LQTS subtypes or taken a broader approach performing an analysis on a cluster of diseases associated with arrhythmogenic or cardiomyopathic causes of sudden cardiac death. The latter approach, clustering several related diseases, may be attractive, it increases the number of cases for analysis and may create a tool that is more relevant for primary care where the question is more likely to be should this patient be investigated or referred, rather than whether they have a specific RD.

### The analytical approach

Predicting rare events poses several challenges. First, there is often little published literature describing the early features of RD, the natural history of the disease and the clinical pathway before diagnosis. Deciding upon exploratory variables for analysis should not only incorporate published literature but also the insights of disease experts and patients affected by the disease.

Second and perhaps most significant, is the relative sparsity of RD cases. Careful consideration should be taken to choose a dataset that is large enough to have sufficient cases whilst remaining representative of the general population into which one envisages the prediction model to be used. In this study, both the dataset CPRD (Gold) with 15 million currently registered patients (CPRD 2023), and the disease, LQTS, a relatively “common” rare disease, were chosen to ensure it would be suitably powered.

Third, the dataset will be significantly imbalanced, that is very few disease outcomes when compared to non-disease outcomes (Feng et al. 2023). In this study we used a case-control design, usually the most appropriate design for rare events, with a propensity score matched control population, this allows a range of covariates to be balanced across the cases and controls especially useful if the population is going to be small, and allows for greater flexibility in the study design (Austin 2011).

Fourth, one should consider how missing data will be handled. Generally given that each RD case is valuable in model development, removing cases if data is missing is not appropriate and multiple imputation, as used in this study, would be preferred to maintain the size of the dataset.

Fifth, managing “sparse data bias”. Multivariate prediction modelling, such as logistic regression, enables one to control simultaneously for multiple confounders. When using such approaches a specific consideration if events are rare is “sparse data bias”, this describes how predictions become increasingly inaccurate as the number of events per variable falls below 20 (Feng et al. 2023; Peduzzi et al. 1996). If sparse data bias is a risk there are a number of statistical approaches that can be used to minimise this (Austin and Steyerberg 2017; Feng et al. 2023).

Sixth, consider what sensitivity analyses are both feasible and desirable. Drug prescriptions and blood investigation results may be suitable to create a cohort of more tightly defined phenotypes. Investigations and prescriptions are typically well recorded in primary care electronic health records.

Seventh, how model performance will be demonstrated. In this study, we show model performance using the metrics: AUC sensitivity, specificity, number needed to test (NNT) $\:\left(NNT=\frac{1}{PPV}\right)\:$(Fig. 2). Choice of evaluation metric is important as an impressively discriminatory AUC may still lead to a far less impressive PPV and therefore NNT when the disease is rare. Ensuring that model performance is described clearly and transparently is important for appropriate decision-making with guidance such as the TRIPOD statement available (Collins et al. 2015).

Eighth, how one will validate the RD prediction model. External validation, that is testing the model in another data set, is usually optimal, however in RD finding a suitable dataset, with both sufficient cases and in a similar clinical setting may not be possible. Internal validation may therefore be appropriate. Internal validation by splitting the dataset into a development and validation set is not recommended, it underpowers model development. Internal validation by bootstrapping, as used in this study, is usually preferred (Collins et al. 2024).

## Electronic supplementary material

Below is the link to the electronic supplementary material.

Supplementary Material 1

Supplementary Material 2

Supplementary Material 3

Supplementary Material 4

Supplementary Material 5

## Data Availability

Data Availability Statement: Data supporting these results are available from the CPRD. Code lists used to perform the analysis are available in supplementary file 1.
