# Supplementary material for: Opportunities and challenges for identifying undiagnosed Rare Disease patients through analysis of primary care records: long QT syndrome as a test case
Source: J Community Genet. 2024 Oct 15;15(6):687–98. doi: 10.1007/s12687-024-00742-7 (PMC11645366; doi:10.1007/s12687-024-00742-7)
Supplement: Supplementary file 2 — Supplementary Material 2 [file 12687_2024_742_MOESM2_ESM.docx]

**LQT associated clinical feature literature search**

**Review question**

**P-** Eligible participants of any age, excluding prenatal patients, from the general population who access both specialist and/or non-specialist clinical settings with a diagnosis of LQTS**.**

**I-** Clinical features to be reported are:

- Clinical features of LQTS.
- Other associated clinical features (eg deafness)

**C-**There are no comparators

**O-** Documentation clinical features

**S-** Included studies will be any reports (case reports, case series, registry publications clinical trial reports or cross-sectional studies) which report clinical features.

Limit to publications that described > 10 patients.

**Databases searched**

The MEDLINE (via Ovid) from 1946 to 28th March 2020, EMBASE database from 1974 to 28th March 2021, Cochrane

Library, CINAHL and PsycINFO databases were electronically searched. The search strategy was adapted for each database.

**Search strings**

EMBASE search string

1. exp long QT syndrome/ 2. long qt syndrome$.ti,ab. 3. LQT$.ti,ab.
4. QTS.ti,ab.

5. or/1-4
6. exp cohort analysis/
7. exp longitudinal study/
8. exp prospective study/
9. exp follow up/
10. cohort$.tw.
11. exp case control study/
12. (case$ and control$).tw.
13. exp case study/
14. (case$ and series).tw.
15. case report/
16. (case$ adj2 report$).tw.
17. (case$ adj2 stud$).tw.
18. (Follow up adj (study or studies)).tw.
19. (observational adj (study or studies)).tw.
20. Cross sectional.tw.
21. Cross-sectional studies/
22. epidemiologic methods/
23. (random$ or placebo$ or single blind$ or double blind$ or triple blind$).ti,ab. 24. (animals not humans).sh.

25. ((comment or editorial or meta-analysis or practice-guideline or review or letter) not randomized controlled trial).pt.

26. (random sampl$ or random digit$ or random effect$ or random survey or random regression).ti,ab. not randomized controlled trial.pt.

27. 24 or 25 or 26 28. 23 not 27
29. or/6-22
30. 28 or 29

31. 5 and 30

MEDLINE search string

1. exp Long QT Syndrome/ 2. long qt syndrome$.ti,ab. 3. LQT$.ti,ab.
4. QTS.ti,ab.

5. or/1-4
6. Epidemiologic studies/
7. exp case control studies/
8. exp cohort studies/
9. Case control.tw.
10. (Follow up adj (study or studies)).tw.
11. (observational adj (study or studies)).tw. 12. Longitudinal.tw.
13. Retrospective.tw.
14. Cross sectional.tw.
15. Cross-sectional studies/
16. cohort$.tw.
17. controlled clinical trial.pt.
18. epidemiologic methods/
19. (case$ and control$).tw.
20. (case$ and series).tw.

21. case reports.pt.

22. (case$ adj2 report$).tw.

23. (case$ adj2 stud$).tw.

24. randomized controlled trial.pt.

25. (random$ or placebo$ or single blind$ or double blind$ or triple blind$).ti,ab.

26. 24 or 25

27. (animals not humans).sh.

28. ((comment or editorial or meta-analysis or practice-guideline or review or letter) not randomized controlled trial).pt.

29. (random sampl$ or random digit$ or random effect$ or random survey or random regression).ti,ab. not randomized controlled trial.pt.

30. 27 or 28 or 29 31. 26 not 30
32. or/6-23,31

PsycINFO search string

1. long qt syndrome$.ti,ab.
2. LQT$.ti,ab.
3. QTS.ti,ab.
4. exp "Arrhythmias (Heart)"/ and exp Syndromes/ 5. epidemiolo*.mp.

6. Case control.tw.
7. (Follow up adj (study or studies)).tw.
8. (observational adj (study or studies)).tw. 9. Longitudinal.tw.
10. Retrospective.tw.
11. Cross sectional.tw.
12. cohort$.tw.
13. (case$ and control$).tw.
14. (case$ and series).tw.
15. (case$ adj2 report$).tw.

16. (case$ adj2 stud$).tw.
17. (random$ or placebo$ or single blind$ or double blind$ or triple blind$).ti,ab. 18. 1 or 2 or 3 or 4
19. 5 or 6 or 7 or 8 or 9 or 10 or 11 or 12 or 13 or 14 or 15 or 16 or 17
20. 18 and 19

EBSCO Search String

http://search.ebscohost.com/login.aspx?direct=true&dbgroup=10805NATSCI&bquery=(epidemiology+OR+(case+ control+study+OR+case- control+study)+OR+(follow+up+stud*)+OR+(cohort+stu*)+OR+(follow+up+stud*)+OR+(follow- up+stud*)+OR+(observational+stud*)+OR+(longtitudinal+stud*)+OR+(retrospective+stud*)+OR+(cross+sectional +stud*)+OR+(cross- sectional+stud*)+OR+cohort%24+OR+(controlled+clinical+trial)+OR+(case+serie*)+OR+(case+report*)+OR+(ran domized+controlled+trials))+AND+(QTS+OR+LQT+OR+((MM+%26quot%3bJervell- Lange+Nielsen+Syndrome%26quot%3b)+OR+(MH+%26quot%3bLong+QT+Syndrome%2b%26quot%3b)+OR+ %26quot%3blong+qt+syndrome%26quot%3b+OR+(MM+%26quot%3bAndersen- Tawil+Syndrome%26quot%3b)))&type=1&searchMode=Standard&site=ehost-live

Full text literature used to create candidate associated clinical features

1. Abu-Zeitone A, Peterson DR, Polonsky B, McNitt S, Moss AJ. Oral contraceptive use and the risk of cardiac events in patients with long QT syndrome. Heart Rhythm. 2014;11(7):1170-5.

2. Al-Hassnan ZN, Al-Fayyadh M, Al-Ghamdi B, Shafquat A, Mallawi Y, Al-Hadeq F, et al. Clinical profile and mutation spectrum of long QT syndrome in Saudi Arabia: The impact of consanguinity. Heart Rhythm. 2017;14(8):1191-9.

3. Ali RH, Zareba W, Moss AJ, Schwartz PJ, Benhorin J, Vincent GM, et al. Clinical and genetic variables associated with acute arousal and nonarousal-related cardiac events among subjects with long QT syndrome. American Journal of Cardiology. 2000;85(4):457-61.

4. Andrsova I, Novotny T, Kadlecova J, Bittnerova A, Vit P, Florianova A, et al. Clinical characteristics of 30 Czech families with long QT syndrome and KCNQ1 and KCNH2 gene mutations: Importance of exercise testing. Journal of Electrocardiology. 2012;45(6):746-51.

5. Auerbach DS, McNitt S, Gross RA, Zareba W, Dirksen RT, Moss AJ. Genetic biomarkers for the risk of seizures in long QT syndrome. Neurology. 2016;87(16):1660-8.

6. Aziz PF, Tanel RE, Zelster IJ, Pass RH, Wieand TS, Vetter VL, et al. Congenital long QT syndrome and 2:1 atrioventricular block: An optimistic outcome in the current era. Heart Rhythm. 2010;7(6):781-5.

7. Aziz PF, Wieand TS, Ganley J, Henderson J, Patel AR, Iyer VR, et al. Genotype- and mutation site-specific QT adaptation during exercise, recovery, and postural changes in children with long-QT syndrome. Circulation: Arrhythmia and Electrophysiology. 2011;4(6):867-73.

8. Berge KE, Haugaa KH, Fruh A, Anfinsen OG, Gjesdal K, Siem G, et al. Molecular genetic analysis of long QT syndrome in Norway indicating a high prevalence of heterozygous mutation carriers. Scandinavian Journal of Clinical & Laboratory Investigation. 2008;68(5):362-8.

9. Blaufox AD, Tristani-Firouzi M, Seslar S, Sanatani S, Trivedi B, Fischbach P, et al. Congenital long QT 3 in the pediatric population. The American Journal Of Cardiology. 2012;109(10):1459-65.

10. Canun S, Perez N, Beirana LG. Andersen syndrome autosomal dominant in three generations. American Journal of Medical Genetics. 1999;85(2):147-56.

11. Chung SK, MacCormick JM, McCulley CH, Crawford J, Eddy CA, Mitchell EA, et al. Long QT and Brugada syndrome gene mutations in New Zealand. Heart Rhythm. 2007;4(10):1306-14.

12. Costa J, Lopes CM, Barsheshet A, Moss AJ, Migdalovich D, Ouellet G, et al. Combined assessment of sex- and mutation-specific information for risk stratification in type 1 long QT syndrome. Heart rhythm. 2012;9(6):892-8.

13. Cuneo B, Swan H, Clur S, Winbo A, Haugaa K, Etheridge S, et al. OC01.03: Incidence of stillbirth is increased in familial LQTS: a retrospective study from eight international centres. Ultrasound in Obstetrics & Gynecology. 2017;50:2-.

14. Czosek RJ, Kaltman JR, Cassedy AE, Shah MJ, Vetter VL, Tanel RE, et al. Quality of Life of Pediatric Patients With Long QT Syndrome. The American Journal Of Cardiology. 2016;117(4):605-10.

15. Dalal A, Czosek RJ, Kovach J, von Alvensleben JC, Valdes S, Etheridge SP, et al. Clinical Presentation of Pediatric Patients at Risk for Sudden Cardiac Arrest. Journal of Pediatrics. 2016;177:191-6.

16. Earle N, Yeo Han D, Pilbrow A, Crawford J, Smith W, Shelling AN, et al. Single nucleotide polymorphisms in arrhythmia genes modify the risk of cardiac events and sudden death in long QT syndrome. Heart Rhythm. 2014;11(1):76-82.

17. Etheridge SP, Sanatani S, Cohen MI, Albaro CA, Saarel EV, Bradley DJ. Long QT syndrome in children in the era of implantable defibrillators. Journal Of The American College Of Cardiology. 2007;50(14):1335-40.

18. Fr¸h A, Siem G, Holmstrˆm H, D¯hlen G, Haugaa KH. The Jervell and Lange-Nielsen syndrome; atrial pacing combined with ﬂ-blocker therapy, a favorable approach in young high-risk patients with long QT syndrome? Heart Rhythm. 2016;13(11):2186-92.

19. Fugate T, 2nd, Moss AJ, Jons C, McNitt S, Mullally J, Ouellet G, et al. Long QT syndrome in African-Americans. Annals of Noninvasive Electrocardiology. 2010;15(1):73-6.

20. Fukushige T, Yoshinaga M, Shimago A, Nishi J, Kono Y, Nomura Y, et al. Effect of age and overweight on the QT interval and the prevalence of long QT syndrome in children. American Journal of Cardiology. 2002;89(4):395-8.

21. Giudicessi JR, Ackerman MJ. Prevalence and potential genetic determinants of sensorineural deafness in KCNQ1 homozygosity and compound heterozygosity. Circulation Cardiovascular Genetics. 2013;6(2):193-200.

22. Goldenberg I, Horr S, Moss AJ, Lopes CM, Barsheshet A, McNitt S, et al. Risk for life-threatening cardiac events in patients with genotype-confirmed long-QT syndrome and normal-range corrected QT intervals. Journal of the American College of Cardiology (JACC). 2011;57(1):51-9.

23. Goldenberg I, Moss AJ, Zareba W, McNitt S, Robinson JL, Qi M, et al. Clinical course and risk stratification of patients affected with the Jervell and Lange-Nielsen syndrome. Journal of Cardiovascular Electrophysiology. 2006;17(11):1161-8.

24. Heradien MJ, Goosen A, Crotti L, Durrheim G, Corfield V, Brink PA, et al. Does Pregnancy Increase Cardiac Risk for LQT1 Patients With the KCNQ1-A341V Mutation? Journal of the American College of Cardiology (JACC). 2006;48(7):1410-5.

25. HyltÈn-Cavallius L, Iepsen EW, Albrechtsen NJW, Svendstrup M, Lubberding AF, Hartmann B, et al. Patients With Long-QT Syndrome Caused by Impaired -Encoded Kv11.1 Potassium Channel Have Exaggerated Endocrine Pancreatic and Incretin Function Associated With Reactive Hypoglycemia. Circulation. 2017;135(18):1705-19.

26. Ilhan A, Tuncer C, Komsuoglu SS, Kali S. Jervell and Lange-Nielsen syndrome: neurologic and cardiologic evaluation. Pediatr Neurol. 1999;21(5):809-13.

27. Johnson JN, Ackerman MJ. Return to play? Athletes with congenital long QT syndrome. British Journal of Sports Medicine. 2013;47(1):28-33.

28. Kelle AM, Bos JM, Etheridge SP, Cannon BC, Bryant RM, Johnson JN, et al. Cardiac transplantation in children and adolescents with long QT syndrome. Heart Rhythm. 2017;14(8):1182-8.

29. Khositseth A, Martinez MW, Driscoll DJ, Ackerman MJ. Syncope in children and adolescents and the congenital long QT syndrome. American Journal of Cardiology. 2003;92(6):746-9.

30. Koponen M, Marjamaa A, Hiippala A, Happonen J-M, Havulinna AS, Salomaa V, et al. Follow-up of 316 molecularly defined pediatric long-QT syndrome patients: clinical course, treatments, and side effects. Circulation Arrhythmia And Electrophysiology. 2015;8(4):815-23.

31. Kutyifa V, Daimee UA, McNitt S, Polonsky B, Lowenstein C, Cutter K, et al. Clinical aspects of the three major genetic forms of long QT syndrome (LQT1, LQT2, LQT3). Annals of Noninvasive Electrocardiology. 2018;23(3):1-.

32. Laitinen P, Fodstad H, Piippo K, Swan H, Toivonen L, Viitasalo M, et al. Survey of the coding region of the HERG gene in long QT syndrome reveals six novel mutations and an amino acid polymorphism with possible phenotypic effects. Human Mutation. 2000;15(6):580-1.

33. Li C, Hu D, Qin X, Li Y, Li P, Liu W, et al. Clinical features and management of congenital long QT syndrome: a report on 54 patients from a national registry. Heart Vessels. 2004;19(1):38-42.

34. MacCormick JM, McAlister H, Crawford J, French JK, Crozier I, Shelling AN, et al. Misdiagnosis of long QT syndrome as epilepsy at first presentation. Annals of Emergency Medicine. 2009;54(1):26-32.

35. Mank-Seymour AR, Richmond JL, Wood LS, Reynolds JM, Fan YT, Warnes GR, et al. Association of torsades de pointes with novel and known single nucleotide polymorphisms in long QT syndrome genes. American Heart Journal. 2006;152(6):1116-22.

36. Mazzanti A, Maragna R, Faragli A, Monteforte N, Bloise R, Memmi M, et al. Gene-Specific Therapy With Mexiletine Reduces Arrhythmic Events in Patients With Long QT Syndrome Type 3. Journal Of The American College Of Cardiology. 2016;67(9):1053-8.

37. Medlock MM, Tester DJ, Will ML, Bos JM, Ackerman MJ. Repeat long QT syndrome genetic testing of phenotype-positive cases: Prevalence and etiology of detection misses. Heart Rhythm. 2012;9(12):1977-82.

38. Millat G, Chevalier B, Restier-Miron L, Da Costa A, Bouvagnet P, Kugener B, et al. Spectrum of pathogenic mutations and associated polymorphisms in a cohort of 44 unrelated patients with long QT syndrome. Clinical Genetics. 2006;70(3):214-27.

39. Mullally J, Goldenberg I, Moss AJ, Lopes CM, Ackerman MJ, Zareba W, et al. Risk of life-threatening cardiac events among patients with long QT syndrome and multiple mutations. Heart Rhythm. 2013;10(3):378-82.

40. Nemec J, Buncova M, Bulkova V, Hejlik J, Winter B, Shen WK, et al. Heart rate dependence of the QT interval duration: differences among congenital long QT syndrome subtypes. Journal of Cardiovascular Electrophysiology. 2004;15(5):550-6.

41. Neyroud N, Maison-Blanche P, Denjoy I, Chevret S, Donger C, Dausse E, et al. Diagnostic performance of QT interval variables from 24-h electrocardiography in the long QT syndrome. European Heart Journal. 1998;19(1):158-65.

42. Olesen MS, Yuan L, Liang B, Hols AG, Nielsen N, Nielsen JB, et al. High prevalence of long QT syndrome-associated SCN5A variants in patients with early-onset lone atrial fibrillation. Circulation: Cardiovascular Genetics. 2012;5(4):450-9.

43. Petko C, Bradley DJ, Tristani-Firouzi M, Cohen MI, Sanatani S, Saarel EV, et al. Congenital long QT syndrome in children identified by family screening. The American Journal Of Cardiology. 2008;101(12):1756-8.

44. Rashba EJ, Zareba W, Moss AJ, Jackson Hall W, Robinson J, Locati EH, et al. Influence of pregnancy on the risk for cardiac events in patients with hereditary long QT syndrome. Circulation. 1998;97(5):451-6.

45. Roberts JD, Krahn AD, Ackerman MJ, Rohatgi RK, Moss AJ, Nazer B, et al. Loss-of-Function Variants: True Monogenic Culprits of Long-QT Syndrome or Proarrhythmic Variants Requiring Secondary Provocation? Circulation: Arrhythmia & Electrophysiology. 2017;10(8):1-11.

46. Rohatgi RK, Sugrue A, Bos JM, Cannon BC, Asirvatham SJ, Moir C, et al. Contemporary Outcomes in Patients With†Long QT Syndrome. Journal Of The American College Of Cardiology. 2017;70(4):453-62.

47. Ruiter JS, Berkenbosch-Nieuwhof K, Van Den Berg MP, Van Dijk R, Middel B, Van Tintelen JP. The importance of the family history in caring for families with long QT syndrome and dilated cardiomyopathy. American Journal of Medical Genetics, Part A. 2010;152(3):607-12.

48. Sakaguchi T, Shimizu W, Itoh H, Noda T, Miyamoto Y, Nagaoka I, et al. Age- and genotype-specific triggers for life-threatening arrhythmia in the genotyped long QT syndrome. J Cardiovasc Electrophysiol. 2008;19(8):794-9.

49. Saprungruang A, Vithessonthi K, La-Orkhun V, Lertsapcharoen P, Khongphatthanayothin A. Clinical presentation and course of long QT syndrome in Thai children. Journal of Arrhythmia. 2015;31(5):296-301.

50. Sauer AJ, Moss AJ, McNitt S, Peterson DR, Zareba W, Robinson JL, et al. Long QT syndrome in adults. Journal Of The American College Of Cardiology. 2007;49(3):329-37.

51. Schwartz PJ, Locati EH, Moss AJ, Crampton RS, Trazzi R, Ruberti U. Left cardiac sympathetic denervation in the therapy of congenital long QT syndrome. A worldwide report. Circulation. 1991;84(2):503-11.

52. Schwartz PJ, Spazzolini C, Crotti L, Bathen J, Amlie JP, Timothy K, et al. The Jervell and Lange-Nielsen syndrome: natural history, molecular basis, and clinical outcome. Circulation. 2006;113(6):783-90.

53. Schwartz PJ, Priori SG, Cerrone M, Spazzolini C, Odero A, Napolitano C, et al. Left cardiac sympathetic denervation in the management of high-risk patients affected by the long-QT syndrome. Circulation. 2004;109(15):1826-33.

54. Schwartz PJ, Spazzolini C, Crotti L. All LQT3 patients need an ICD: True or false? Heart Rhythm. 2009;6(1):113-20.

55. Seth R, Moss AJ, McNitt S, Zareba W, Andrews ML, Qi M, et al. Long QT syndrome and pregnancy. Journal of the American College of Cardiology. 2007;49(10):1092-8.

56. Sherman J, Tester DJ, Ackerman MJ. Targeted mutational analysis of ankyrin-B in 541 consecutive, unrelated patients referred for long QT syndrome genetic testing and 200 healthy subjects. Heart Rhythm. 2005;2(11):1218-23.

57. Shimizu W, Moss AJ, Wilde AA, Towbin JA, Ackerman MJ, January CT, et al. Genotype-phenotype aspects of type 2 long QT syndrome. Journal of the American College of Cardiology. 2009;54(22):2052-62.

58. Swan H, Toivonen L, Viitasalo M. Rate adaptation of QT intervals during and after exercise in children with congenital long QT syndrome. European Heart Journal. 1998;19(3):508-13.

59. Takenaka K, Ai T, Shimizu W, Kobori A, Ninomiya T, Otani H, et al. Exercise stress test amplifies genotype-phenotype correlation in the LQT1 and LQT2 forms of the long-QT syndrome. Circulation. 2003;107(6):838-44.

60. Tester DJ, Will ML, Haglund CM, Ackerman MJ. Compendium of cardiac channel mutations in 541 consecutive unrelated patients referred for long QT syndrome genetic testing. Heart Rhythm. 2005;2(5):507-17.

61. Tester DJ, Will ML, Haglund CM, Ackerman MJ. Effect of Clinical Phenotype on Yield of Long QT Syndrome Genetic Testing. Journal of the American College of Cardiology. 2006;47(4):764-8.

62. Tomas M, Napolitano C, De Giuli L, Bloise R, Subirana I, Malovini A, et al. Polymorphisms in the NOS1AP Gene Modulate QT Interval Duration and Risk of Arrhythmias in the Long QT Syndrome. Journal of the American College of Cardiology. 2010;55(24):2745-52.

63. Viitasalo M, Oikarinen L, Swan H, Vaananen H, Jarvenpaa J, Hietanen H, et al. Effects of beta-blocker therapy on ventricular repolarization documented by 24-h electrocardiography in patients with type 1 long-QT syndrome. Journal of the American College of Cardiology. 2006;48(4):747-53.

64. Vincent GM, Timothy KW, Leppert M, Keating M. The spectrum of symptoms and QT intervals in carriers of the gene for the long-QT syndrome. N Engl J Med. 1992;327(12):846-52.

65. Wang F, Liu J, Hong L, Liang B, Graff C, Yang Y, et al. The phenotype characteristics of type 13 long QT syndrome with mutation in KCNJ5 (Kir3.4-G387R). Heart Rhythm. 2013;10(10):1500-6.

66. Wilde AA, Moss AJ, Kaufman ES, Shimizu W, Peterson DR, Benhorin J, et al. Clinical Aspects of Type 3 Long-QT Syndrome: An International Multicenter Study. Circulation. 2016;134(12):872-82.

67. Winbo A, Diamant UB, Rydberg A, Jensen SM, Stattin EL. Age and origin of the Y111C/KCNQ1 founder mutation-a major cause of the long-QT syndrome in Sweden. Annals of Hematology. 2011;1):e14.

68. Yasuda K, Hayashi G, Horie A, Taketani T, Yamaguchi S. Clinical and electrophysiological features of Japanese pediatric long QT syndrome patients with KCNQ1 mutations. Pediatrics International. 2008;50(5):611-4.

69. Yoon G, Oberoi S, Tristani-Firouzi M, Etheridge SP, Quitania L, Kramer JH, et al. Andersen-Tawil syndrome: prospective cohort analysis and expansion of the phenotype. Am J Med Genet A. 2006;140(4):312-21.

70. Yuan L, Olesen MS, Holst A, Nielsen N, Liang B, Nielsen JB, et al. High prevalence of long QT syndrome associated SCN5A variants in patients with early-onset lone atrial fibrillation. Journal of Interventional Cardiac Electrophysiology. 2012;33 (3):330.

71. Zareba W, McNitt S, Moss AJ. Risk stratification in LQTS patients with QTC of 441-500MS. Heart Rhythm. 2009;1):S124.

72. Zellerhoff S, Pistulli R, Monnig G, Hinterseer M, Beckmann BM, Kobe J, et al. Atrial arrhythmias in long-QT syndrome under daily life conditions: A nested case control study. Journal of Cardiovascular Electrophysiology. 2009;20(4):401-7.

73. Zhang C, Kutyifa V, McNitt S, Zareba W, Goldenberg I, Moss AJ. Identification of Low-Risk Adult Congenital LQTS Patients. Journal Of Cardiovascular Electrophysiology. 2015;26(8):853-8.

74. Zhang L, Benson DW, Tristani-Firouzi M, Ptacek LJ, Tawil R, Schwartz PJ, et al. Electrocardiographic features in Andersen-Tawil syndrome patients with KCNJ2 mutations: characteristic T-U-wave patterns predict the KCNJ2 genotype. Circulation. 2005;111(21):2720-6.

75. Zhang L, Vincent GM, Baralle M, Baralle FE, Anson BD, Benson DW, et al. An intronic mutation causes long QT syndrome. Journal of the American College of Cardiology. 2004;44(6):1283-91.
