# Supplementary material for: Opportunities and challenges for identifying undiagnosed Rare Disease patients through analysis of primary care records: long QT syndrome as a test case
Source: J Community Genet. 2024 Oct 15;15(6):687–98. doi: 10.1007/s12687-024-00742-7 (PMC11645366; doi:10.1007/s12687-024-00742-7)
Supplement: Supplementary file 4 — Supplementary Material 4 [file 12687_2024_742_MOESM4_ESM.pdf]

4 Missing data continuous variables

Variable	Missing data cases & controls (8970)	Missing data cases (1495)
BMI	1937 (21.6%)	126 (8.4%)
BP	1451 (16.2%)	58 (3.9%)
Pulse	7338 (81.8%)	966 (64.6%)
Potassium level	4993 (55.7%)	430 (28.8%)
Calcium level	7104 (79.2%)	935 (62.5%)
