# Supplementary material for: Opportunities and challenges for identifying undiagnosed Rare Disease patients through analysis of primary care records: long QT syndrome as a test case
Source: J Community Genet. 2024 Oct 15;15(6):687–98. doi: 10.1007/s12687-024-00742-7 (PMC11645366; doi:10.1007/s12687-024-00742-7)
Supplement: Supplementary file 5 — Supplementary Material 5 [file 12687_2024_742_MOESM5_ESM.pdf]

3 Previous iterations of model

xi: logistic case_control ht_flag ameno_flag stillbirth_misc_flag rharth_flag ibs_flag epilepsy _flag migra_flag corrtachy_brady brady_flag tachy_flag white_nonwhite rharth_flag hoarse_flag dizzi_flag collapse_flag aneurysm_dissect_flag aort_valv_flag mit_valv_flag non_spec_valv_flag palp_flag hf_flag cad_flag stroke_flag af_flag corrmean_hypocalcemia hypocal_flag corrmean_hypokalaemia hypokal_flag i.bb_category i.bmi_cat

AUC 0.7453

xi: logistic case_control ht_flag stillbirth_misc_flag ibs_flag epilepsy_flag corrtachy_brady brady_flag tachy_flag white_nonwhite dizzi_flag collapse_flag aneurysm_dissect_flag aort_valv_flag mit_valv_flag non_spec_valv_flag palp_flag hf_flag cad_flag stroke_flag af_flag corrmean_hypocalcemia hypocal_flag corrmean_hypokalaemia hypokal_flag i.bb_category i.bmi_cat

AUC 0.7452

xi: logistic case_control ht_flag stillbirth_misc_flag ibs_flag epilepsy_flag corrtachy_brady brady_flag tachy_flag white_nonwhite dizzi_flag collapse_flag mit_valv_flag palp_flag hf_flag cad_flag stroke_flag af_flag corrmean_hypocalcemia hypocal_flag corrmean_hypokalaemia hypokal_flag i.bb_category i.bmi_cat

AUC 0.7449

xi: logistic case_control ht_flag stillbirth_misc_flag ibs_flag epilepsy_flag corrtachy_brady brady_flag tachy_flag white_nonwhite dizzi_flag collapse_flag mit_valv_flag palp_flag hf_flag cad_flag stroke_flag af_flag corrmean_hypocalcemia hypocal_flag corrmean_hypokalaemia hypokal_flag i.bb_category i.bmi_cat

AUC 0.7443

xi: logistic case_control ht_flag ibs_flag epilepsy_flag corrtachy_brady brady_flag white_nonwhite dizzi_flag collapse_flag mit_valv_flag palp_flag af_flag corrmean_hypocalcemia hypocal_flag corrmean_hypokalaemia hypokal_flag i.bb_category i.bmi_cat

AUC 0.7431

xi: logistic case_control ht_flag ibs_flag epilepsy_flag corrtachy_brady brady_flag white_nonwhite dizzi_flag collapse_flag mit_valv_flag palp_flag af_flag corrmean_hypocalcemia corrmean_hypokalaemia i.bb_category i.bmi_cat

AUC=0.7429

xi: logistic case_control ht_flag ibs_flag epilepsy_flag brady_flag white_nonwhite dizzi_flag collapse_flag mit_valv_flag palp_flag af_flag corrmean_hypocalcemia corrmean_hypokalaemia i.bb_category i.bmi_cat

AUC= 0.7413

xi: logistic case_control ht_flag ibs_flag epilepsy_flag brady_flag white_nonwhite dizzi_flag collapse_flag palp_flag af_flag corrmean_hypocalcemia corrmean_hypokalaemia i.bb_category

AUC= 0.7380

xi: logistic case_control ht_flag ibs_flag epilepsy_flag brady_flag white_nonwhite collapse_flag palp_flag af_flag corrmean_hypocalcemia corrmean_hypokalaemia i.bb_category

AUC= 0.7347

Multiple imputed

xi: logistic case_control ibs_flag epilepsy_flag corrtachy_brady brady_flag tachy_flag white_nonwhite dizzi_flag collapse_flag palp_flag af_flag cad_flag bmi_fp_1 bmi_fp_2 cal_fp_1 cal_fp_2 potass_fp_1 potass_fp_2 smoktype.

0.7266 (0.7128, 0.7404)

xi: logistic case_control ibs_flag epilepsy_flag corrtachy_brady brady_flag tachy_flag white_nonwhite dizzi_flag collapse_flag palp_flag af_flag bmi_fp_1 bmi_fp_2 cal_fp_1 cal_fp_2 potass_fp_1 potass_fp_2 smoktype

0.7233 (0.7094 0.7373)

xi: logistic case_control   ibs_flag epilepsy_flag corrtachy_brady brady_flag tachy_flag dizzi_flag collapse_flag   palp_flag af_flag bmi_fp_1 bmi_fp_2 cal_fp_1 cal_fp_2 potass_fp_1 potass_fp_2 smoktype

0.7090 (0.6946 0.7232)

xi: logistic case_control ht_flag ibs_flag epilepsy_flag corrtachy_brady brady_flag tachy_flag white_nonwhite dizzi_flag collapse_flag mit_valv_flag palp_flag af_flag corrmean_hypocalcemia corrmean_hypokalaemia hypokal_flag i.bb_category ameno_flag aneurysm_dissect_flag aort_valv_flag bmi_cat cad_flag cereban_flag corrbmi corrdiabp corrmean_calcium   corrmean_potass corrmean_pulse corrsysbp endo_flag gender hf_flag hoarse_flag hypermob_flag migra_flag non_spec_valv_flag pad_flag pcos_flag pneumo_flag pul_valv_flag rharth_flag sah_flag sicklecell_flag smoktype stillbirth_misc_flag stroke_flag subfert_flag tri_valv_flag

AUC 0.7297

AIC 7359.517

BIC 7714.599

xi: logistic case_control ht_flag ibs_flag epilepsy_flag corrtachy_brady brady_flag tachy_flag white_nonwhite dizzi_flag collapse_flag mit_valv_flag palp_flag af_flag corrmean_hypocalcemia   i.bb_category   brady_flag bmi_cat cad_flag collapse_flag corrbmi corrdiabp corrmean_calcium corrmean_hypocalcemia

corrmean_hypokalaemia corrmean_potass corrtachy_brady epilepsy_flag hf_flag ibs_flag smoktype tachy_brady white_nonwhite

AUC 0.7287

AIC 7330.091

BIC 7514.734

xi: logistic case_control ht_flag ibs_flag epilepsy_flag corrtachy_brady brady_flag tachy_flag white_nonwhite dizzi_flag collapse_flag mit_valv_flag palp_flag af_flag corrmean_hypocalcemia i.bb_category bmi_cat cad_flag corrbmi corrdiabp corrmean_calcium corrmean_potass hf_flag smoktype

AUC 0.7288

AIC 7328.489

BIC 7506.03
